# Outcomes After Kidney Transplantation in Antiglomerular Basement Membrane Disease

**DOI:** 10.1016/j.ekir.2025.06.021

**Published:** 2025-06-18

**Authors:** Priscille Traversat, Marine Dekervel, Christophe Masset, Dominique Bertrand, Léonard Golbin, Philippe Gatault, Antoine Thierry, Emilie Cornec-Le Gall, Maïté Jaureguy, Cyrille Garrouste, Dany Anglicheau, Valérie Chatelet, Sophie Caillard, Anna Duval, Jean-Philippe Rerolle, Martin Planchais, Agnès Duveau, Fabien Duthe, Jean-François Augusto, Benoît Brilland

**Affiliations:** 1 Service de Néphrologie-Dialyse-Transplantation, CHU Angers, Angers, France; 2 Institut de Transplantation-Urologie-Néphrologie, Nantes University Hospital, Nantes, France; 3 Service de Néphrologie-Dialyse-Transplantation, CHU de Rouen, Rouen, France; 4 Service de Néphrologie-Dialyse-Transplantation, CHU de Rennes, Rennes, France; 5 Service de Néphrologie-Dialyse-Transplantation, CHU de Tours, Tours, France; 6 Service de Néphrologie-Dialyse-Transplantation, CHU de Poitiers, Poitiers, France; 7 Université de Brest, Inserm, UMR 1078, GGB, CHU Brest, Centre de référence MARHEA, Brest, France; 8 Service de Néphrologie-Dialyse-Transplantation, CHU d’Amiens, Amiens, France; 9 Service de Néphrologie-Dialyse-Transplantation, CHU de Clermont-Ferrand, Clermont-Ferrand, France; 10 Service des Maladies du rein et du métabolisme, Hôpital Necker, Assistance Publique-Hôpitaux de Paris, Paris, France; 11 Service de Néphrologie-Dialyse-Transplantation, CHU de Caen, Caen, France; 12 Service de Néphrologie-Dialyse-Transplantation, CHU de Strasbourg, Strasbourg, France; 13 Service de Néphrologie-Dialyse-Transplantation, CHU de Limoges, Limoges, France; 14 Univ Angers, Nantes Université, Inserm, CNRS, CRCI2NA, SFR ICAT, Angers, France

**Keywords:** GBM, glomerulonephritis, Goodpasture, kidney transplantation, outcomes, vasculitis

## Abstract

**Introduction:**

Goodpasture disease, or antiglomerular basement membrane (anti-GBM) disease, is a rare autoimmune disorder that often leads to end-stage kidney disease (ESKD). Although kidney transplantation (KT) is the preferred treatment, concerns exist about disease recurrence and graft outcomes in patients with GBM-associated glomerulonephritis (GBM-GN). This study aimed to evaluate posttransplant outcomes in patients with GBM-GN compared with matched controls.

**Methods:**

This retrospective, multicenter study included 100 patients with anti-GBM who received KT between 2005 and 2023 in 13 French transplant centers, matched with 200 control recipients. We compared the incidence of delayed graft function (DGF), graft failure, relapse, acute rejection, and death between groups and analyzed risk factors using multivariable models.

**Results:**

No significant differences in DGF incidence (22% vs. 19%, *P* = 0.5), graft survival (87% vs. 88% at 5 years, *P* = 0.4), or patient survival (93% vs. 89% at 5 years, *P* = 0.4) were found between patients with GBM-GN and controls. Patients with GBM-GN tended to have lower risk of acute rejection (hazard ratio [HR] = 0.51, 95% confidence interval: 0.25–1.02, *P* = 0.055). Only 1 patient with GBM-GN (1%) experienced disease relapse. Although patients with GBM-GN were waitlisted and transplanted later than controls, specific transplant timing was not associated with improved outcomes.

**Conclusion:**

KT in patients with GBM-GN offers comparable outcomes to other nephropathies in the current era. Disease relapse is rare, even in the few patients with detectable antibodies pretransplantation. The lower incidence of acute rejection in the GBM-GN group warrants further investigation. These findings support KT as a viable option in patients with GBM-GN, though specific pre- and posttransplant monitoring is advised.

## Graphical abstract

**Figure:**
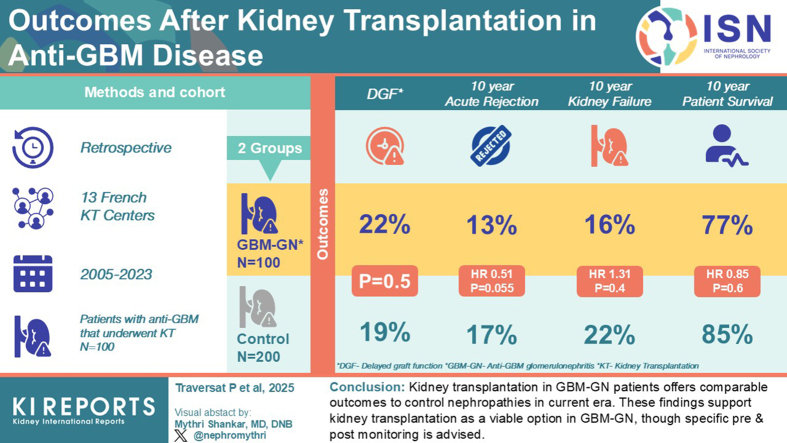

Goodpasture disease, also known as anti-GBM disease, is a rare autoimmune disorder characterized by the presence of circulating anti-GBM antibodies that target the basement membranes of kidneys and lungs. Clinically, it presents with rapidly progressive GN (GBM-GN), often accompanied by pulmonary hemorrhage. Kidney involvement is present in approximately 80% to 90% of cases,^1^ manifesting as hematuria, proteinuria, and progressive kidney failure. GBM-GN can then lead to significant morbidity, with 50% to 70% of patients progressing to ESKD despite early intervention, and particularly if the diagnosis is delayed or if renal function is severely impaired at presentation.^2^^,^^3^ The prognosis for renal recovery is largely determined by the severity of kidney injury and the promptness of treatment initiation.^3^

As in other kidney diseases, KT has emerged as the preferred treatment option for patients with GBM-GN with ESKD and is considered to offer superior overall survival and quality of life compared with maintenance dialysis, in analogy with other nephropathies, but without specific data. In the context of Goodpasture disease, transplantation carries specific risks as follows: antibodies can reemerge, and the disease can relapse; in addition, pretransplantation immunosuppression carries its specific long-term effects that adds up to those of posttransplantation regimens.

Despite the importance of optimal timing for KT in patients with GBM-GN, current guidelines are limited. Based on multiple reports of disease recurrence (up to 50%) in patients who have detectable anti-GBM antibodies at the time of KT, Kidney Disease: Improving Global Outcomes^4^ and the Canadian Society of Transplantation^5^ recommend a minimum of 6 months of complete serological remission before considering KT. There is currently no European recommendation on the management of these patients before or after transplantation. Although patient and graft survivals previously appeared similar for patients with GBM-GN when compared with other transplant recipients,^6^, ^7^, ^8^, ^9^ optimal timing for KT and factors associated with posttransplant outcomes (relapse, rejection, graft survival, and patient survival) have been poorly studied, mostly on small-scale studies (or macroscopic registry studies), conducted in an earlier era, and with controversial results. To address these critical knowledge gaps, we conducted an observational, retrospective, multicenter study, in the era of modern immunosuppressive regimens, in which we aimed to describe the incidence of DGF, graft failure, relapse, acute rejection, and death of patients with GBM-GN compared with a control group, and to investigate the risk factors associated with these events.

## Methods

### Selection of Patients

This retrospective study included adult patients who received a kidney transplant in 13 French transplant centers as follows: 12 centers (Spiesser Group) that share a common transplant database (ASTRE)^10^^,^^11^ and 1 additional center (Nantes DIVAT^12^ [Données Informatisées et VAlidées en Transplantation] cohort collaborators). These databases were used to identify patients who received a kidney transplant between 2005 and 2023, for whom ESKD was caused by GBM-GN (cases) or other reasons (controls).

### Selection of Cases

In native kidney, GBM-GN diagnosis was based on active renal involvement (active urine sediment with hematuria, proteinuria and/or impaired renal function) and often associated with pulmonary involvement (intraalveolar hemorrhage), and/or anti-GBM antibodies in serum. In most cases, it was confirmed by a renal biopsy showing GN with linear deposition of anti-GBM antibodies. When kidney biopsy was not performed, anti-GBM positivity was mandatory. One case was included twice because the patient received 2 transplants over time.

### Selection of Controls

Each GBM-GN case was systematically matched with 2 controls of the same center. Controls were selected from the same databases (ASTRE and DIVAT) among all patients who received a kidney transplant during the study period (2005–2023) for ESKD because of any cause other than GBM-GN. The matching process prioritized the following criteria in hierarchical order: (i) same transplant center (mandatory), (ii) same sex when possible, (iii) recipient’s age within ±5 years when possible, and (iv) transplant period within ±5 years when possible (Supplementary Figure S1). When multiple potential controls met these criteria, selection was performed by minimizing differences of age and period as much as possible. This matching strategy ensured that controls were representative of the general kidney transplant population at each center during the study period.

### Data Collection

For all patients, cases or controls, pre- and posttransplant clinical, biological, histological, and treatment data (Table 1) were manually retrieved from individual medical records. Glomerular filtration rate was estimated using the Chronic Kidney Disease Epidemiology Collaboration research group equation.^13^ For kidney function evaluation after transplantation, patients returned to dialysis were assigned a creatinine of 500 μmol/l and an estimated glomerular filtration rate of 5 ml/min. For cases, anti-GBM antibodies were assessed in indirect immunofluorescence and/or quantitative enzyme immunoassay.

**Table 1:** Description of patients

Characteristics	*n*	Overall, *N* = 300	CTRL, *n* = 200	GBM-GN, *n* = 100	*P*-value
Baseline characteristics					
Male sex, *n* (%)	300	170 (57%)	112 (56%)	58 (58%)	0.7
BMI (kg/m^2^)	298	24.4 (4.4)	24.9 (4.6)	23.5 (4.0)	0.008
Hypertension, *n* (%)	288	243 (84%)	166 (88%)	77 (77%)	0.012
Diabetes, *n* (%)	298	42 (14%)	33 (17%)	9 (9.0%)	0.072
Kidney disease, *n* (%)	300				
GBM-GN		-	-	100 (100%)	
ADPKD		-	39 (20%)	-	
Immunological (not GBM disease)		-	38 (19%)	-	
Genetic (not ADPKD)		-	26 (13%)	-	
Diabetic		-	16 (8.0%)	-	
Urologic		-	12 (6.0%)	-	
Vascular		-	14 (7.0%)	-	
Others		-	14 (7.0%)	-	
Unknown		-	41 (21%)	-	
Presentation at vasculitis diagnosis					
Age (yrs)	97	-	-	43 (18)	
Kidney involvement, *n* (%)	100	-	-	100 (100%)	
Creatinine (μmol/l)	63	-	-	956 (555)	
Need for KRT within 30 d, *n* (%)	87	-	-	74 (85%)	
Proteinuria (g/g)	49	-	-	3.12 (3.75)	
Hematuria, *n* (%)	47	-	-	46 (98%)	
Kidney biopsy, *n* (%)	198	106 (54%)	31 (26%)	75 (93%)	<0.001
Lung involvement, *n* (%)	81	-	-	32 (40%)	
Immunological findings					
Presence of anti-GBM abs, *n* (%)	84	-	-	75 (89%)	
Presence of ANCA (IIF or EIA), *n* (%)	66	-	-	14 (21%)	
Anti-PR3	56	-	-	2 (3.6%)	
Anti-MPO	56	-	-	5 (8.9%)	
Therapeutic management					
Induction remission therapy		-	-		
Plasma exchange, *n* (%)	90	-	-	73 (81%)	
Methylprednisolone pulses, *n* (%)	93	-	-	86 (92%)	
Prednisone, *n* (%)	92	-	-	83 (90%)	
Cyclophosphamide, *n* (%)	93	-	-	77 (83%)	
Rituximab, *n* (%)	92	-	-	3 (3.3%)	
Maintenance therapy		-	-		
Prednisone, *n* (%)	71	-	-	48 (68%)	
Cyclophosphamide, *n* (%)	75	-	-	10 (13%)	
Rituximab, *n* (%)	75	-	-	1 (1.3%)	
Azathioprine, *n* (%)	76	-	-	8 (11%)	
Mycophenolic acid, *n* (%)	75	-	-	7 (9.3%)	
Outcomes before KT					
Relapse	90	-	-	2 (2.2%)	
Preemptive transplantation	300	39 (13%)	34 (17%)	5 (5.0%)	0.004
Kidney transplantation					
Status at KT					
Age (yrs)	300	50 (16)	51 (16)	50 (16)	0.5
First transplantation	299	250 (84%)	166 (83%)	84 (84%)	0.9
Calculated PRA	206	25 (36)	24 (35)	27 (38)	0.5
Serological status					
Anti-GBM abs positive (considering IIF+ or EIA+), *n* (%)	49	-	-	3 (6.1%)	
Anti-GBM abs positive (considering IIF only), *n* (%)	43	-	-	3 (7.0%)	
Anti-GBM abs positive (considering EIA only), *n* (%)	42	-	-	2 (4.8%)	
Transplantation procedure					
Donor age (yrs)	282	53 (17)	54 (17)	51 (18)	0.3
Deceased donor, *n* (%)	297	259 (87%)	169 (86%)	90 (90%)	0.3
ABO incompatibility, *n* (%)	262	4 (1.5%)	3 (1.7%)	1 (1.1%)	>0.9
Donor creatinine (μmol/l)	262	82 (45)	83 (48)	80 (38)	0.6
Cold ischemia time (h)	293	15 (8)	15 (8)	14 (7)	0.5
HLA-mismatches (total)	296	4.19 (1.66)	4.16 (1.67)	4.26 (1.66)	0.6
HLA-A	296	1.15 (0.66)	1.13 (0.67)	1.17 (0.64)	0.6
HLA-B	296	1.39 (0.66)	1.37 (0.66)	1.43 (0.66)	0.5
HLA-DR	296	0.95 (0.69)	0.95 (0.69)	0.95 (0.71)	>0.9
HLA-DQ	247	0.84 (0.69)	0.85 (0.71)	0.83 (0.64)	0.9
Immunosuppressive regimen					
Induction therapy					
Antithymocyte globulin, *n* (%)	298	103 (35%)	62 (31%)	41 (41%)	0.10
Basiliximab, *n* (%)	297	184 (62%)	129 (65%)	55 (55%)	0.079
Prednisone, *n* (%)	297	276 (93%)	185 (93%)	91 (93%)	>0.9
Maintenance regimen					
Calcineurin inhibitors, *n* (%)	299	296 (99%)	196 (98%)	100 (100%)	0.6
Mycophenolic acid, *n* (%)	298	290 (97%)	191 (96%)	99 (99%)	0.3
Prednisone, *n* (%)	282	210 (74%)	131 (70%)	79 (82%)	0.030
mTOR inhibitors, *n* (%)	294	41 (14%)	26 (13%)	15 (15%)	0.6
Azathioprine, *n* (%)	293	19 (6.5%)	12 (6.2%)	7 (7.1%)	0.7
Outcomes after KT					
Follow-up duration (mo)	300	86 (43, 132)	76 (42, 123)	97 (46, 145)	0.14
DGF, *n* (%)	286	56 (20%)	35 (19%)	21 (22%)	0.5
Allograft failure, *n* (%)	299	54 (18%)	37 (19%)	17 (17%)	0.7
Relapses					
All, *n* (%)	299	-	-	1 (1.0%)	-
With kidney involvement, *n* (%)	299	-	-	1 (1.0%)	-
Rejection, *n* (%)	294	56 (19%)	43 (22%)	13 (13%)	0.074
Acute rejection (AR), *n* (%)	294	45 (15%)	34 (17%)	11 (11%)	0.2
TCMR, *n* (%)	294	29 (9.9%)	22 (11%)	7 (7.1%)	0.3
ABMR, *n* (%)	293	17 (5.8%)	14 (7.2%)	3 (3.1%)	0.2
Mixed AR, *n* (%)	300	8 (2.7%)	6 (3.0%)	2 (2.0%)	0.7
Chronic rejection	291	16 (5.5%)	14 (7.2%)	2 (2.1%)	0.069
Death	300	49 (16%)	30 (15%)	19 (19%)	0.4

ABMR, antibody-mediated rejection; abs, antibodies; ADPKD, autosomal dominant polycystic kidney disease; ANCA, antineutrophil cytoplasmic autoantibodies; anti-MPO, antimyeloperoxidase, antimyeloperoxisae antibodies; Anti-PR3, antiproteinase 3 antibodies; AR, acute rejection; BMI, body mass index; CTRL, controls; DGF, delayed graft function; EIA, enzyme immunoassays; GBM, glomerular basement membrane; GBM-GN, glomerular basement membrane disease-associated glomerulonephritis; HLA, human leukocyte antigen; IIF, indirect immunofluorescence; KRT, kidney replacement therapy; KT, kidney transplantation; mTOR, mammalian target of rapamycin; PRA, panel reactive antibody; TCMR, T-cell–mediated rejection.

### Definitions and Outcomes of Interest

Posttransplant outcomes were defined as follows:

1. DGF was defined as previously^14^: requirement for ≥ 1 dialysis session within 7 days of transplantation.
2. Kidney allograft failure (ESKD) was defined by return to dialysis or retransplantation.
3. GBM disease relapse was defined as previously^15^: renal flares were diagnosed based on biopsy evidence of necrotizing crescentic GN with linear IgG deposition along the GBM in indirect immunofluorescence and increased serum creatinine associated with nephritic urinary sediment, in the absence of other causes of renal allograft dysfunction. Extrarenal flares were diagnosed based on an episode of pulmonary hemorrhage without evidence of infection. In the control group, recurrence of initial nephropathy was not considered.
4. Acute rejection was defined as in Banff classification,^16^ acute antibody-mediated rejection and acute T-cell–medicated rejection were recorded but considered together for subsequent analyses. Borderline rejections were considered as part of acute T-cell–medicated rejection. Chronic rejection events were also recorded.

### Statistical Analysis

Continuous variables were described with mean ± SD, except for delays between events, presented as median (1st–3rd quartiles). Categorical variables were described with counts and percentages. Data were compared using the *t* test for continuous variables, and χ^2^ test (or Fisher exact test if necessary) for categorical variables.

For the estimation of patient survival, Kaplan–Meier analyses were performed, and survival curves were compared with a log-rank test. The cumulative incidence of the above-mentioned outcomes of interest were evaluated with death as a competing event using the cumulative incidence competing risk method.^17^ Cumulative incidence curves were compared using Gray’s test.

Cox regression analyses were used for the following: (i) adjust event-free survival when comparing cases and controls and (ii) perform multivariable analyses to examine factors associated with outcomes in the GBM-GN group. Multivariate Cox regression analysis included all parameters with *P* < 0.1 in the univariable analysis. To simplify the multivariable models, the number of variables was limited using manual step-by-step backward selection with a removal criterion of *P* > 0.1. HR with 95% confidence intervals were reported. A logistic regression analysis was performed to examine factors associated with DGF in the GBM-GN group. Multivariable analysis was carried out as described above. Odds ratios with 95% confidence intervals were reported. No imputation of missing data was performed. Statistical analyses were performed using R version 4.0. All tests were 2-sided, and a *P*-value < 0.05 was considered statistically significant.

### Ethical Issues

Our cohort has been declared and authorized by the “Commission Nationale Informatique et Libertés” (agreement number 202200131 / ar23-0077v0). This study was approved by the local ethics committee of Angers University Hospital (2022-175). Owing to the nature of the study and in accordance with French law, the patients’ written consent was not mandatory.

## Results

### Description of GBM-GN Cases

A total of 100 patients received a KT for ESKD secondary to GBM-GN. Patients’ characteristics are summarized in Table 1. Patients with GBM-GN were diagnosed between 1985 and 2021. The mean age at diagnosis was 43 ± 18 years, two-thirds of patients (58/100, 58%) were males, and mostly (75/84, 89%) found with anti-GBM antibodies at diagnosis. Most of them (75/81, 93%) had a biopsy proven GBM-GN. All of them had anti-GBM antibodies and/or biopsy proven GBM-GN. A fifth of them (14/66, 21%) also had coexistence of antineutrophil cytoplasmic autoantibodies (ANCA). Before KT, nearly none (2/90, 2.2%) of them had relapsed. The mean age at KT was 50 ± 16 years, and patients were transplanted after a median waiting time of 11 (4–21) months. Five patients (5/100, 5%) were transplanted preemptively, and the median time in dialysis before KT was 31 (22–45) months for the others (Supplementary Figure S2). Donor source was mostly deceased donor (90/100, 90%). There was 1 ABO (1/91, 1.1%) incompatible transplantation; the mean calculated panel reactive antibody was 27% ± 38%. At the time of transplantation, anti-GBM antibodies status (indirect immunofluorescence or enzyme immunoassay) was evaluated in 49 patients (49/100, 49%). It was positive in at least 1 technique for 3 of them (3/49, 6.1%), without specific pretransplant therapeutic intervention. Median follow-up after KT was 97 (46–145) months.

### Description of Control Patients

Characteristics of the 200 controls included in the study are summarized in Table 1. Overall, 100%, 91%, 84%, and 78% of cases were well-matched with controls regarding transplant center, year of transplantation, sex, and recipient age, respectively (Supplementary Figure S1).

Mean age at KT was 51 ± 16 years. Thirty-four patients (34/200, 17%) were transplanted preemptively, and the median time in dialysis before KT was 19 (6–36) months for the other patients (Supplementary Figure S2). Donor source was mostly deceased donors (169/197, 86%). There were 3 cases (1.7%) of ABO incompatibility; the mean calculated panel reactive antibody was 24% ± 35%. Causes of ESKD before KT were mostly led by autosomal dominant polycystic kidney disease (39/200, 20%), immunological causes (38/200, 18%), other genetic causes (26/200, 13%), and unknown causes (41/200, 21%). Median follow-up after KT was 76 ([42–123) months.

### Kidney Function and Survival

#### DGF and Kidney Function Evolution

There was no significant difference in the incidence of DGF between groups, involving 21/97 (22%) and 35/189 (19%) patients in the GBM-GN and control group, respectively (*P* = 0.5, Table 1). Factors associated with the occurrence of DGF, in the GBM-GN group, in univariable analysis are described in Supplementary Table S1. In multivariable analysis, donor age (odds ratio = 1.04 [1.01–1.08], *P* = 0.022) and cold ischemia time (odds ratio = 1.12 [1.03–1.23], *P* = 0.013) were the sole factors associated with DGF (Supplementary Table S1).

**Figure 1:**
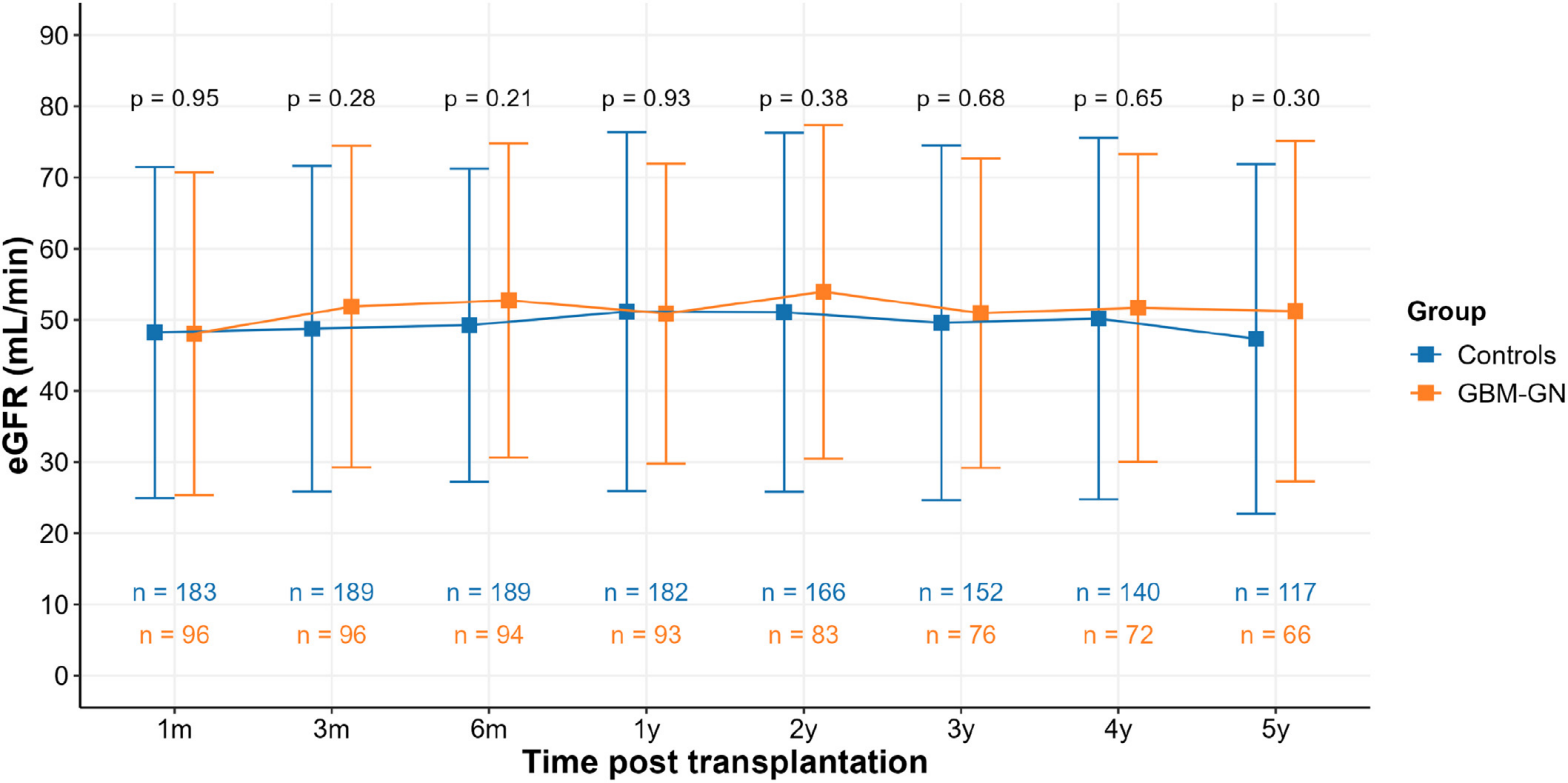
eGFR evolution after kidney transplantation. Data are represented as mean ± SD. eGFR, estimated glomerular filtration rate; GBM-GN, glomerular basement membrane disease-associated glomerulonephritis.

There was no significant difference in kidney function evolution over time; when considering the whole cohort, creatinine levels were 150 ± 75 and 158 ± 88 μmol/l at 6 months and 1 year, respectively; and estimated glomerular filtration rate levels were 51 ± 21 and 53 ± 24 ml/min at 6 months and 1 year, respectively. Similar values of estimated glomerular filtration rate were observed over the first 5 years of follow-up in both groups (Figure 1).

#### Graft Survival

**Figure 2:**
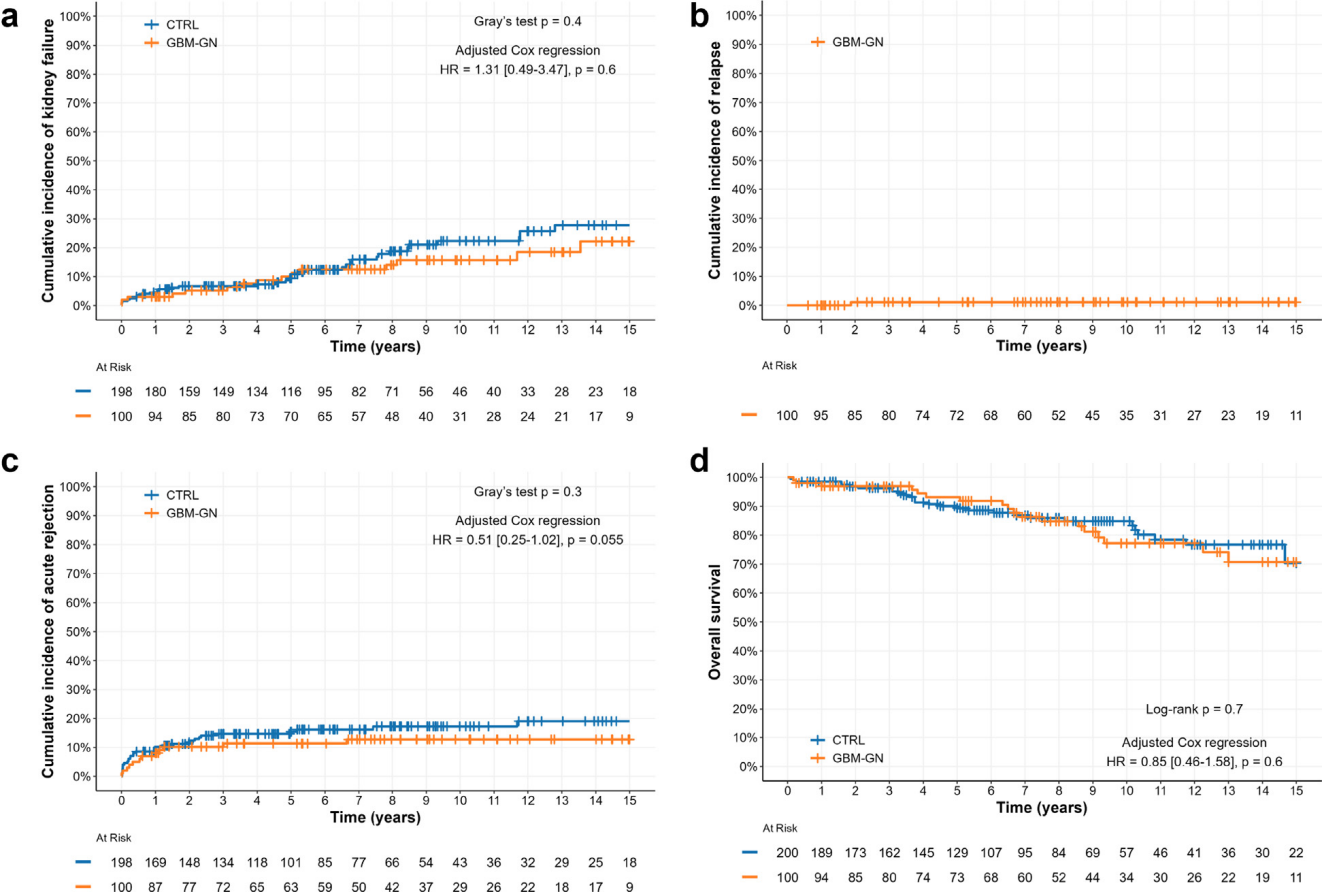
Event-free survival after kidney transplantation. (a) Cumulative incidence of ESKD, (b) relapse, and (c) acute rejection. (a and c) Cumulative incidence curves were compared with Gray’s test. (d) Overall survival (Kaplan-Meier curves were compared with log-rank test). CTRL, control; ESKD, end-stage kidney disease; GBM-GN, glomerular basement membrane disease-associated glomerulonephritis; HR, hazards ratio.

A total of 54 graft failures occurred during follow-up as follows: 17/100 (17%) and 37/199 (19%) in GBM-GN and control group, respectively. In univariable analysis, there was no significant difference in the cumulative incidence of graft loss when comparing the GBM-GN group with the control group (3.0% vs. 4.6% at 1 year, 10% vs. 9.4% at 5 years, and 16% vs. 22% at 10 years, *P* = 0.4, Figure 2a). After adjustment, no difference was found in graft survival between these groups (HR = 1.31 [0.49–3.47], *P* = 0.6, Supplementary Table S2).

In the GBM-GN group, in multivariable analysis, donor age (HR = 1.03 [1.00–1.06], *P* = 0.085) and chronic rejection (HR = 16.7 [3.38–82.4], *P* < 0.001) were associated with graft loss (Table 2). Maintenance therapy with mycophenolate mofetil (HR = 0.15 [0.02–1.30], *P* = 0.085) was associated with graft survival.

**Table 2:** Factors associated with graft failure (in the GBM-GN cohort)

Characteristics	Univariable					Multivariable (simplified)				
Baseline characteristics	*N*	Event *n*	HR	95% CI	*P*-value	*N*	Event *n*	HR	95% CI	*P*-value
Male sex (vs. female)	100	17	1.54	0.54–4.40	0.4					
BMI (kg/m^2^)	99	16	1.03	0.91–1.17	0.6					
Hypertension (vs. no)	100	17	1.04	0.34–3.21	>0.9					
Diabetes (vs. no)	100	17	1.67	0.38–7.37	0.5					
Presentation at vasculitis diagnosis										
Age (yrs)	97	17	1.01	0.98–1.04	0.6					
Kidney involvement (vs. no)	100	17	Inf.	Inf.	-					
Lung involvement (vs. no)	81	12	0.62	0.18–2.09	0.4					
Immunological findings										
Presence of anti-GBM abs (vs. no)	84	11	0.98	0.12–7.78	>0.9					
Presence of ANCA (vs. no)	66	8	3.65	0.79–16.8	0.1					
Therapeutic management										
Induction remission therapy										
Plasma exchange (vs. no)	90	15	1.25	0.28–5.58	0.8					
Methylprednisolone pulses (vs. no)	93	15	Inf.	Inf.	-					
Prednisone (vs. no)	92	15	Inf.	Inf.	-					
Cyclophosphamide (vs. no)	93	16	1.25	0.28–5.53	0.8					
Rituximab (vs. no)	92	16	Inf.	Inf.	-					
Maintenance therapy										
Prednisone (vs. no)	71	9	0.55	0.15–2.05	0.4					
Cyclophosphamide (vs. no)	75	9	0.84	0.11–6.74	0.9					
Rituximab (vs. no)	75	9	0.00	0.00–Inf	>0.9					
Azathioprine (vs. no)	76	9	2.12	0.44–10.3	0.4					
Mycophenolic acid (vs. no)	75	9	Inf.	Inf.	-					
Kidney transplantation										
Status at KT										
Age (yrs)	100	17	1.02	0.99–1.06	0.14					
First transplantation	100	17	0.41	0.14–1.16	0.092					
Preemptive transplantation	100	17	1.26	0.16–9.61	0.8					
Calculated PRA	69	9	1.00	0.99–1.02	0.8					
Serological status (positive vs. negative)^a^	49	7	Inf.	Inf.	-					
Transplantation procedure										
Donor age (yrs)	92	16	1.03	1.00–1.06	0.081	89	16	1.03	1.00–1.06	0.085
Deceased donor (vs. no)	100	17	1.99	0.26–15.1	0.5					
Donor creatinine (/50 μmol/l increment)	90	14	0.89	0.41–1.93	0.8					
Cold ischemia time (h)	99	16	1.09	1.02–1.18	0.014					
HLA-mismatches (total)	98	16	0.85	0.63–1.15	0.3					
HLA-A	98	16	0.69	0.31–1.49	0.3					
HLA-B	98	16	0.64	0.32–1.28	0.2					
HLA-DR	98	16	0.58	0.28–1.19	0.14					
HLA-DQ	83	15	1.34	0.59–3.02	0.5					
Immunosuppressive regimen										
Induction therapy										
Anti-thymocyte globulin (vs. no)	100	17	1.35	0.52–3.50	0.5					
Basiliximab (vs. no)	100	17	0.72	0.28–1.87	0.5					
Prednisone (vs. no)	98	17	Inf.	Inf.	-					
Maintenance regimen										
Calcineurin inhibitors (vs. no)	100	17	Inf.	Inf.	-					
Mycophenolic acid (vs. no)	100	17	0.11	0.01–0.87	0.036	89	16	0.15	0.02–1.30	0.085
Prednisone (vs. no)	96	16	0.57	0.20–1.65	0.3					
mTOR inhibitors (vs. no)	98	16	1.38	0.39–4.89	0.6					
Azathioprine (vs. no)	98	16	0.55	0.07–4.23	0.6					
Outcomes after KT										
DGF (vs. no)	97	16	2.05	0.70–6.04	0.2					
Allograft failure (vs. no)	-	-	-	-	-					
Rejection (vs. no)	98	17	1.99	0.65–6.13	0.2					
Acute rejection (AR) (vs. no)	98	17	1.71	0.49–5.97	0.4					
TCMR (vs. no)	98	17	0.64	0.08–4.81	0.7					
ABMR (vs. no)	98	17	2.80	0.37–21.4	0.3					
Mixed AR (vs. no)	100	17	4.55	0.59–34.9	0.14					
Chronic rejection	97	17	12.1	2.63–55.3	0.001	89	16	16.7	3.38–82.4	<0.001
Death	-	-	-	-	-					

ABMR, antibody-mediated rejection; abs, antibodies; ADPKD, autosomal dominant polycystic kidney disease; ANCA, antineutrophil cytoplasmic autoantibodies; AR, acute rejection; BMI, body mass index; CI, confidence interval; DGF, delayed graft function; EIA, enzyme immunoassays; GBM, glomerular basement membrane; GBM-GN, glomerular basement membrane disease-associated glomerulonephritis; HLA, human leukocyte antigen; HR, hazard ratio; IIF, indirect immunofluorescence; KRT, kidney replacement therapy; KT, kidney transplantation; mTOR, mammalian target of rapamycin; PRA, panel reactive antibody; TCMR, T-cell–mediated rejection.

When there were not enough data in some subgroup, Cox regression model could not converge (result is given as “Inf.”).

a Anti-GBM serological status at the time of KT encompasses both IIF and EIA methods.

### Relapses

Only 1 patient with GBM-GN (11/99, 1%) relapsed after transplantation (Figure 2b). This case was challenging, because the patient was negative for anti-GBM antibodies at the time of diagnosis (biopsy proven), transplantation, and relapse. Relapse occurred 22 months after KT, it was both renal (biopsy proven) and extrarenal (diffuse alveolar hemorrhage) and the patient rapidly required dialysis. This case has been extensively reported elsewhere.^18^

Interestingly, 3 patients (of the 49 with available antibody assessment: 6.1%) were transplanted with detectable anti-GBM antibodies, none of them relapsed after a follow-up duration of 12, 84, and 173 months, respectively. Given the small number of antibody-positive patients, this subgroup analysis should be interpreted with caution.

### Acute Rejection–Free Survival

Because there were few heterogenous events (*n* = 16/291), we did not analyze chronic rejection data and focused on acute rejection events. A total of 45 acute rejection events occurred during the follow-up, considering T-cell–medicated rejection, antibody-mediated rejection, and mixed acute rejection together: 11/99 (11%) and 34/195 (17%) events occurred in the GBM-GN and control groups, respectively (Table 1).

In univariable analysis, there was no significant difference in the cumulative incidence of acute rejection when the GBM-GN group was compared with the control group (8.0% vs. 10% at 1 year, 11% vs. 15% at 5 years, and 13% vs. 17% at 10 years, *P* = 0.3, Figure 2c). However, in multivariable analysis, patients with GBM-GN were at lower risk of acute rejection; that did not reach statistical significance (HR = 0.51 [0.25–1.02], *P* = 0.055, Supplementary Table S3).

In the GBM-GN group, in multivariable analysis, anti-GBM antibody positivity at diagnosis (HR = 0.11 [0.03–0.50], *P* = 0.004) and donor creatinine (HR = 1.71 [0.93–3.13] per 50 μmol/l increments, *P* = 0.084) were the sole factors associated (or tending to be associated) with acute rejection (Table 3).

**Table 3:** Factors associated with acute rejection (in the GBM-GN cohort)

Characteristics	Univariable					Multivariable (simplified)				
Baseline characteristics	*N*	Event *n*	HR	95% CI	*P*-value	*N*	Event *n*	HR	95% CI	*P*-value
Male sex (vs. female)	97	11	3.30	0.71–15.3	0.13					
BMI (kg/m^2^)	96	11	1.01	0.88–1.16	> 0.9					
Hypertension (vs. no)	97	11	0.34	0.11–1.13	0.078					
Diabetes (vs. no)	97	11	2.31	0.50–10.7	0.3					
Presentation at vasculitis diagnosis										
Age (yrs)	95	11	0.98	0.95–1.01	0.3					
Kidney involvement (vs. no)	97	11	Inf.	Inf.	-					
Lung involvement (vs. no)	79	8	0.96	0.23–4.01	> 0.9					
Immunological findings										
Presence of anti-GBM abs (vs. no)	82	8	0.18	0.04–0.77	0.021	74	8	0.11	0.03–0.50	0.004
Presence of ANCA (vs. no)	65	7	0.64	0.08–5.32	0.7					
Therapeutic management										
Induction remission therapy										
Plasma exchange (vs. no)	88	9	0.71	0.15–3.42	0.7					
Methylprednisolone pulses (vs. no)	91	10	Inf.	Inf.	-					
Prednisone (vs. no)	90	10	0.87	0.11–6.89	0.9					
Cyclophosphamide (vs no)	91	10	1.83	0.23–14.4	0.6					
Rituximab (vs. no)	90	10	Inf.	Inf.	-					
Maintenance therapy										
Prednisone (vs. no)	69	7	1.20	0.23–6.20	0.8					
Cyclophosphamide (vs. no)	73	8	2.54	0.51–12.6	0.3					
Rituximab (vs. no)	73	8	Inf.	Inf.	-					
Azathioprine (vs. no)	74	8	2.96	0.60–14.7	0.2					
Mycophenolic acid (vs. no)	73	8	1.40	0.17–11.4	0.8					
Kidney transplantation										
Status at KT										
Age (yrs)	97	11	0.98	0.94–1.02	0.3					
First transplantation	97	11	0.88	0.19–4.07	0.9					
Preemptive transplantation	97	11	2.20	0.28–17.2	0.5					
Calculated PRA	68	7	1.00	0.98–1.02	0.9					
Serological status (positive vs. negative)^a^	47	7	Inf.	Inf.	-					
Transplantation procedure										
Donor age (yrs)	89	11	0.98	0.95–1.01	0.2					
Deceased donor (vs. no)	97	11	1.14	0.15–8.93	0.9					
Donor creatinine (per 50 μmol/l increment)	88	11	1.65	1.03–2.64	0.038	74	8	1.71	0.93–3.13	0.084
Cold ischemia time (h)	96	11	0.95	0.87–1.03	0.2					
HLA-mismatches (total)	96	11	0.91	0.63–1.30	0.6					
HLA-A	96	11	0.42	0.16–1.06	0.067					
HLA-B	96	11	0.70	0.30–1.66	0.4					
HLA-DR	96	11	0.99	0.42–2.34	>0.9					
HLA-DQ	81	11	1.38	0.53–3.60	0.5					
Immunosuppressive regimen										
Induction therapy										
Antithymocyte globulin (vs. no)	97	11	0.49	0.13–1.87	0.3					
Basiliximab (vs. no)	97	11	1.52	0.45–5.20	0.5					
Prednisone (vs. no)	96	10	0.68	0.09–5.34	0.7					
Maintenance regimen										
Calcineurin inhibitors (vs. no)	97	11	Inf.	Inf.	-					
Mycophenolic acid (vs. no)	97	11	Inf.	Inf.	-					
Prednisone (vs. no)	93	9	1.84	0.23–14.7	0.6					
mTOR inhibitors (vs. no)	95	10	1.35	0.29–6.38	0.7					
Azathioprine (vs. no)	95	10	Inf.	Inf.	-					
Outcomes after KT										
DGF (vs. no)	94	9	1.00	0.21–4.82	> 0.9					
Allograft failure (vs. no)	97	11	1.84	0.49–6.94	0.4					
Rejection (vs. no)	-	-	-	-	-					
Acute rejection (AR) (vs. no)	-	-	-	-	-					
TCMR (vs. no)	-	-	-	-	-					
ABMR (vs. no)	-	-	-	-	-					
Mixed AR (vs. no)	-	-	-	-	-					
Chronic rejection	-	-	-	-	-					
Death	-	-	-	-	-					

-, not applicable; ABMR, antibody-mediated rejection; abs, antibodies; ADPKD, autosomal dominant polycystic kidney disease; ANCA, antineutrophil cytoplasmic autoantibodies; AR, acute rejection; BMI, body mass index; CI, confidence interval; DGF, delayed graft function; EIA, enzyme immunoassays; GBM, glomerular basement membrane; GBM-GN, glomerular basement membrane disease-associated glomerulonephritis; HLA, human leukocyte antigen; HR, hazard ratio; IIF, indirect immunofluorescence; KRT, kidney replacement therapy; KT, kidney transplantation; mTOR, mammalian target of rapamycin; PRA, panel reactive antibody; TCMR, T-cell–mediated rejection.

When there was not enough data in some subgroup, Cox regression model could not converge (result is given as "Inf.").

a Anti-GBM serological status at the time of KT encompass both IIF and EIA methods.

### Patient Survival

A total of 49 deaths occurred during the follow-up: 19/100 (19%) and 30/200 (15%) in the GBM-GN and control group, respectively. In univariable analysis, there was no significant difference in patient survival when comparing the GBM-GN group with the control group (97% vs. 99% at 1 year, 93% vs. 89% at 5 years, and 77% vs. 85% at 10 years; *P* = 0.4; Figure 2d). After adjustment, no difference was found in GBM-GN and control patients’ graft survival (HR = 0.85 [0.46–1.58], *P* = 0.6, Supplementary Table S4).

In the GBM-GN group, in multivariable analysis, age at diagnosis (HR = 1.09 [1.05–1.14], *P* < 0.001), number of total mismatches (HR = 0.64 [0.47–0.86], *P* = 0.004) and allograft failure (HR = 6.17 [1.82–21.0], *P* = 0.004) were associated with death (Table 4).

**Table 4:** Factors associated with death (in the GBM-GN cohort)

Characteristics	Univariable					Multivariable (simplified)				
Baseline characteristics	*N*	Event *n*	HR	95% CI	*P*-value	*N*	Event *n*	HR	95% CI	*P*-value
Male sex (vs. female)	100	19	1.82	0.65–5.07	0.3					
BMI (kg/m^2^)	99	19	1.12	1.01–1.25	0.030					
Hypertension (vs. no)	100	19	1.21	0.40–3.65	0.7					
Diabetes (vs. no)	100	19	0.57	0.08–4.26	0.6					
Presentation at vasculitis diagnosis										
Age (yrs)	97	18	1.06	1.02–1.09	< 0.001	95	17	1.09	1.05–1.14	<0.001
Kidney involvement (vs. no)	100	19								
Lung involvement (vs. no)	81	15	0.31	0.09–1.12	0.074					
Immunological findings										
Presence of anti-GBM abs (vs. no)	84	15	0.44	0.12–1.58	0.2					
Presence of ANCA (vs. no)	66	14	2.84	0.83–9.70	0.1					
Therapeutic management										
Induction remission therapy										
Plasma exchange (vs. no)	90	17	1.44	0.33–6.33	0.6					
Methylprednisolone pulses (vs. no)	93	16	Inf.	Inf.	-					
Prednisone (vs. no)	92	16	1.15	0.15–8.85	0.9					
Cyclophosphamide (vs. no)	93	17	0.81	0.23–2.84	0.7					
Rituximab (vs. no)	92	17	Inf.	Inf.	-					
Maintenance therapy										
Prednisone (vs no)	71	15	0.39	0.14–1.07	0.068					
Cyclophosphamide (vs no)	75	14	0.38	0.05–3.10	0.4					
Rituximab (vs no)	75	14	Inf.	Inf.	-					
Azathioprine (vs no)	76	14	Inf.	Inf.	-					
Mycophenolic acid (vs no)	75	14	0.72	0.09–5.55	0.8					
Kidney transplantation										
Status at KT										
Age (yrs)	100	19	1.08	1.04–1.12	< 0.001					
First transplantation	100	19	0.94	0.27–3.29	> 0.9					
Preemptive transplantation	100	19	Inf.	Inf.	-					
Calculated PRA	69	13	1.00	0.99–1.02	0.9					
Serological status (positive vs negative)^a^	49	9	Inf.	Inf.	-					
Transplantation procedure										
Donor age (yrs)	92	18	1.06	1.03–1.09	< 0.001					
Deceased donor (vs. no)	100	19	0.47	0.16–1.42	0.2					
Donor creatinine (per 50 μmol/L increment)	90	17	0.73	0.31–1.70	0.5					
Cold ischemia time (h)	99	18	1.02	0.95–1.09	0.6					
HLA-mismatches (total)	98	18	0.77	0.59–1.01	0.056	95	17	0.64	0.47–0.86	0.004
HLA-A	98	18	0.46	0.22–0.94	0.034					
HLA-B	98	18	0.62	0.32–1.20	0.2					
HLA-DR	98	18	1.01	0.52–1.97	>0.9					
HLA-DQ	83	15	0.54	0.22–1.32	0.2					
Immunosuppressive regimen										
Induction therapy										
Antithymocyte globulin (vs. no)	100	19	0.79	0.31–2.03	0.6					
Basiliximab (vs. no)	100	19	1.26	0.50–3.21	0.6					
Prednisone (vs. no)	98	18	1.26	0.17–9.53	0.8					
Maintenance regimen										
Calcineurin inhibitors (vs. no)	100	19								
Mycophenolic acid (vs. no)	100	19	Inf.	Inf.	-					
Prednisone (vs. no)	96	18	1.44	0.42–5.02	0.6					
mTOR inhibitors (vs. no)	98	18	1.37	0.40–4.75	0.6					
Azathioprine (vs. no)	98	18	Inf.	Inf.	-					
Outcomes after KT										
DGF (vs. no)	97	19	2.44	0.95–6.31	0.065					
Allograft failure (vs. no)	100	19	2.95	1.16–7.54	0.023	95	17	6.17	1.82–21.0	0.004
Rejection (vs. no)	98	18	0.59	0.14–2.57	0.5					
Acute rejection (AR) (vs. no)	98	18	0.37	0.05–2.76	0.3					
TCMR (vs. no)	98	18	0.48	0.06–3.65	0.5					
ABMR (vs. no)	98	18	0.00	0.00–Inf	> 0.9					
Mixed AR (vs. no)	100	19	0.00	0.00–Inf	> 0.9					
Chronic rejection	97	18	2.85	0.37–21.7	0.3					
Death	-	-	-	-	-					

-, not applicable; ABMR, antibody-mediated rejection; abs, antibodies; ADPKD, autosomal dominant polycystic kidney disease; ANCA, antineutrophil cytoplasmic autoantibodies; AR, acute rejection; BMI, body mass index; CI, confidence interval; DGF, delayed graft function; EIA, enzyme immunoassays; GBM, glomerular basement membrane; GBM-GN, glomerular basement membrane disease-associated glomerulonephritis; HLA, human leukocyte antigen; HR, hazard ratio; IIF, indirect immunofluorescence; KRT, kidney replacement therapy; KT, kidney transplantation; mTOR, mammalian target of rapamycin; PRA, panel reactive antibody; TCMR, T-cell mediated rejection.

When there were not enough data in some subgroup, Cox regression model could not converge (result is given as "Inf.").

a Anti-GBM serological status at the time of KT encompasses both IIF and EIA methods.

### Delays Between Events and Association With Outcomes

Although the time between waitlisting and KT was similar between GBM-GN and control patients (11 vs. 14 months, *P* = 0.11), the time between ESKD and KT (31 vs. 19 months, *P* < 0.001), and between ESKD and waitlisting (16 vs. 7 months, *P* < 0.001) was higher for patients with GBM-GN (Supplementary Figure S1B and C and Supplementary Table S5).

We then studied the impact of delays on the previously described events. When focusing on the GBM-GN group, in univariable analysis, there was no clear trend of an impact of the various considered delays on graft survival, acute rejection-free survival, and patient survival (Supplementary Figure S3). Some analyses were, however, lacking power because of the low number of patients with short delays (< 6 months or < 12 months) between these time points.

### Characteristics and Outcomes of Patients With GBM-GN According to ANCA Status

Among the 66 GBM-GN transplant recipients with known ANCA status at diagnosis, 14 (21%) were ANCA positive. These patients were significantly older both at diagnosis (58 ± 12 vs. 41 ± 17 years; *P* < 0.001) and at transplantation (62 ± 12 vs. 46 ± 16 years; *P* < 0.001) than their ANCA-negative counterparts, and they exhibited pulmonary involvement less often (8.3% vs. 42%; *P* = 0.042). Although limited by the sample size, ANCA-positive patients experienced a trend for lower graft and patient survival (Supplementary Figure S4). No association was found between ANCA status at KT and allograft (HR = 2.39 [0.48–11.8], *P* = 0.3) or patient (HR = 1.71 [0.47–6.15, *P* = 0.4) survival after adjustment on age at transplantation.

## Discussion

This study is, to the best of our knowledge, one of the largest retrospective analyses to date on KT outcomes in patients with GBM-GN compared with a matched control group.^6^, ^7^, ^8^^,^^15^ We found no significant difference in kidney function evolution, DGF occurrence, graft survival, or overall survival between GBM-GN and control recipients. There was a trend for a lower acute rejection rate in patients with GBM-GN and we only identified 1 case of relapse. Chronic rejection and donor age were major factors influencing graft survival, whereas positive anti-GBM antibodies at diagnosis seemed protective against acute rejection in the GBM-GN group. Patients with GBM-GN were waitlisted and transplanted later than controls, but no specific transplant timing was associated with improved outcomes. Lastly, no therapeutic strategy, either pre- or posttransplant, was associated with the outcomes studied.

The absence of a significant difference in graft and patient survival between GBM-GN and control groups suggests that modern immunosuppressive regimens and KT practices can offer outcomes in patients with GBM-GN that are comparable with those of patients with other nephropathies. Despite the initial concerns regarding disease recurrence or specific adverse effects linked to pretransplant immunosuppression, our findings are reassuring. This is in line with previous smaller studies.^6^^,^^8^^,^^9^ In our cohort, the 5-year graft and overall survival rate for patients with anti-GBM-GN reached 87% and 93% respectively, congruent with recent studies.^8^^,^^15^

In our cohort, relapse was very rare (1%) and occurred in a patient who was antibody negative at the time of diagnosis, transplantation and relapse. The pathophysiology behind this relapse may involve cryptic or nondetectable low-level antibody production or other immune mechanisms not captured by current diagnostic methods. The low relapse rate in our cohort aligns with recent smaller studies.^8^^,^^15^ Previous studies have reported much higher relapse rates, as high as 50% in patients transplanted with detectable anti-GBM antibodies; however, most of these studies were conducted before the introduction of contemporary immunosuppressive regimens.^19^ Without questioning the rationale behind the current recommendations from Kidney Disease: Improving Global Outcomes^4^ and Canadian guidelines,^5^ it is interesting to note that none of the 3 patients transplanted with positive antibodies at the time of KT relapsed in our cohort. However, this observation should be interpreted with considerable caution, given the following: (i) the small number of patients with available antibody data at transplantation (*n* = 49); (ii) the even smaller number with positive antibodies (*n* = 3); and (iii) the fact that current clinical practice, guided by existing guidelines, may have influenced patient selection (e.g., patients with higher antibody titers may have been systematically excluded from transplantation) and timing of transplantation. Therefore, though our data suggest that the actual risk of relapse may be lower than historically reported in the contemporary era, they do not provide sufficient evidence to modify current practices or question existing guidelines.

Surprisingly, our multivariate analysis showed that patients with Goodpasture's disease tended to have a lower risk of acute rejection compared with the control group (HR = 0.51, *P* = 0.055). In addition, when focusing on the GBM-GN group alone, patients with positive anti-GBM antibody at diagnosis were at lower risk of acute rejection (HR = 0.11, *P* = 0.004). A possible explanation could be that these patients received more intense immunosuppression (more thymoglobulin in induction therapy and more steroid in maintenance regimen) because of their underlying autoimmune disease, which might have reduced the risk of rejection. This finding however raises questions about the potential immune tolerance or protective mechanisms developed in some patients with GBM-GN with high initial antibody titers, warranting further investigation.

In our cohort, the interval from ESKD onset to waitlist inscription and to KT was longer among patients with GBM-GN, largely reflecting standard practice of awaiting at least 6 months of serological remission before listing, combined with the often severe, multiorgan course of disease that necessitates prolonged medical optimization. In addition, caution on the part of clinicians and transplant teams—exacerbated by the rarity of anti-GBM disease—and slower referral pathways may further extend time to waitlisting. Importantly, though limited by the sample size and event number, these delays did not seem to adversely affect graft or patient survival.

Our study has several limitations, mostly due to its retrospective design that may have introduced inherent biases, including selection bias and data availability. However, if the ASTRE database was used to identify patients transplanted with GBM-GN, all data were manually and carefully gathered, on each patient's record on a detailed scale. In addition, only a small rate of GBM-GN relapse after KT, precluding stronger statistical analysis. Another limitation lies in the incomplete antibody data at the time of KT for many patients (half of them), which could have provided a more robust analysis of the relationship between serological remission and outcomes, especially regarding patients with positive anti-GBM antibodies at the time of KT. Further, very few patients received a KT within 12 months, or even within 24 months from GBM-GN diagnosis, precluding any specific analysis on this delay. Finally, this study did not assess the quality of life, an important factor in KT recipients.

Nonetheless, this is the largest French reported experience with KT for GBM-GN, and one of the largest studies on this topic so far. Our study provides valuable insights into the posttransplant outcomes of GBM-GN in the modern era. By including a matched control group and conducting multivariable analyses, we were able to adjust for many potential confounders, providing a comprehensive understanding of the factors influencing graft and patient survival in patients with GBM-GN. In addition, our long-term follow-up allowed us to capture a wide range of clinically significant outcomes, offering a detailed picture of the posttransplant course in this specific patient population.

In conclusion, this study demonstrates that KT in patients with GBM-GN offers graft and patient survival outcomes comparable with those of other nephropathies. Although our findings uphold existing guidelines mandating serological remission before transplantation, incomplete titer data prevent firm conclusions about the predictive power of antibody levels alone. Accordingly, future studies with comprehensive antibody monitoring and investigation of antibody-independent drivers of relapse are warranted. Clinicians can use these results to counsel patients with GBM-GN more confidently about the safety and efficacy of KT in the current therapeutic landscape. Further prospective studies are needed to refine serological and clinical monitoring strategies posttransplant.

## Disclosure

All the authors declared no competing interests.

## Supplementary Material

Supplementary File (PDF) **Figure S1.** Matching between GBM-GN cases and control patients. **Figure S2.** Distribution of delays (time between diagnosis, ESKD, waitlisting and kidney transplantation). **Figure S3.** Impact of delays on event-free survival after kidney transplantation (in the GBM-GN subgroup). **Figure S4.** Event-free survival after kidney transplantation according to ANCA status at GBM-GN diagnosis. **Table S1.** Factors associated with DGF (in the GBM-GN cohort). **Table S2.** Factors associated with graft failure (in the whole cohort). **Table S3.** Factors associated with acute rejection (in the whole cohort). **Table S4.** Factors associated with death (in the whole cohort). **Table S5.** Distribution of delays between diagnosis, ESKD, waitlisting and kidney transplantation.

## Footnotes

Supplementary File (PDF)
**Figure S1.** Matching between GBM-GN cases and control patients. **Figure S2.** Distribution of delays (time between diagnosis, ESKD, waitlisting and kidney transplantation). **Figure S3.** Impact of delays on event-free survival after kidney transplantation (in the GBM-GN subgroup). **Figure S4.** Event-free survival after kidney transplantation according to ANCA status at GBM-GN diagnosis. **Table S1.** Factors associated with DGF (in the GBM-GN cohort). **Table S2.** Factors associated with graft failure (in the whole cohort). **Table S3.** Factors associated with acute rejection (in the whole cohort). **Table S4.** Factors associated with death (in the whole cohort). **Table S5.** Distribution of delays between diagnosis, ESKD, waitlisting and kidney transplantation.
