# Supplementary material for: Outcomes After Kidney Transplantation in Antiglomerular Basement Membrane Disease
Source: Kidney Int Rep. 2025 Jun 18;10(9):3150–63. doi: 10.1016/j.ekir.2025.06.021 (PMC12446932; doi:10.1016/j.ekir.2025.06.021)
Supplement: Supplementary File (PDF) — Figure S1. Matching between GBM-GN cases and control patients. Figure S2. Distribution of delays (time between diagnosis, ESKD, waitlisting and kidney transplantation). Figure S3. Impact of delays on event-free survival after kidney transplantation (in the GBM-GN subgroup). Figure S4. Event-free survival after kidney transplantation according to ANCA status at GBM-GN diagnosis. Table S1. Factors associated with DGF (in the GBM-GN cohort). Table S2. Factors associated with graft failure (in the whole cohort). Table S3. Factors associated with acute rejection (in the whole cohort). Table S4. Factors associated with death (in the whole cohort). Table S5. Distribution of delays between diagnosis, ESKD, waitlisting and kidney transplantation. [file mmc1.pdf]

Outcomes after kidney transplantation in anti-GBM disease

Priscille Traversat[1*], Marine Dekervel[1*], Christophe Masset[2], Dominique Bertrand[3], Léonard Golbin[4], Philippe Gatault[5], Antoine Thierry[6], Emilie Cornec-Le Gall[7], Maïté Jaureguy[8], Cyrille Garrouste[9], Dany Anglicheau[10], Valérie Chatelet[11], Sophie Caillard[12], Anna Duval[12], Jean-Philippe Rerolle[13], Martin Planchais[1], Agnès Duveau[1], Fabien Duthe[6], Jean-François Augusto[1,14], Benoît Brilland[1,14].

Supplementary Material.

Supplementary Figures (PDF)

Supplementary Tables (PDF)

Supplementary Figure and Table legends.

Supplementary Figure S1 – Matching between GBM-GN cases and control patients.

Matching quality regarding center (A), year of transplantation (B), sex (C) and recipient age (D). GBM-GN cases were considered well-matched with controls if they came from the same center (A), were transplanted during the same period (± 5 years, B), were of the same sex (C) and within the same age range (± 5 years, D).

Abbreviations: GBM-GN, glomerular basement membrane disease-associated glomerulonephritis.

Supplementary Figure S2 – Distribution of delays (time between diagnosis, ESKD, waitlisting and kidney transplantation).

A. Schematic of delays between GBM-GN diagnosis (when applicable), ESKD, waitlisting, and kidney transplantation in the GBM-GN (red) and control (green) groups.

B. Delays between GBM-GN diagnosis (when applicable), ESKD, waitlisting, and kidney transplantation in the GBM-GN (red) and control (green) groups ; as continuous variable.

C. Delays between GBM-GN diagnosis (when applicable), ESKD, waitlisting, and kidney transplantation in the GBM-GN (red) and control (green) groups ; as 3 categories variable.

Abbreviations: GBM-GN, glomerular basement membrane disease-associated glomerulonephritis; CTRL, control; Dg, diagnosis; ESKD, end-stage kidney disease; KT, kidney transplantation; WL, waitlisting.

Supplementary Figure S3 – Impact of delays on event-free survival after kidney transplantation (in the GBM-GN subgroup).

Cox proportional-hazards analysis of the impact of timing intervals on event-free survival after kidney transplantation in the GBM-GN subgroup. Delays were categorized in three ways: Continuous (per 6-month increment), in blue; dichotomous (> 6, > 12, or > 24 months, with the shortest interval as reference), in green; three-category (< 12, 12–36, > 36 months, with the middle category as reference), in red.

Hazard ratios (95% confidence intervals) are shown for each delay type and for each outcome (allograft failure, acute rejection, and death). The association between delays and relapses is not plotted due to the single event observed (n = 1). A dashed horizontal line indicates HR = 1; dotted vertical lines separate delay-type groups. Note that some 95% CI error bars span the full

height of the plot, reflecting extremely wide or unbounded intervals arising from small subgroup sizes.

Abbreviations: Dg, diagnosis; ESKD, end-stage kidney disease; GBM-GN, glomerular basement membrane disease-associated glomerulonephritis; KT, kidney transplantation; WL, waitlisting.

Supplementary Figure S4 – Event-free survival after kidney transplantation according to ANCA status at GBM-GN diagnosis.

Survival free of ESKD (A), relapse (B), acute rejection (D) or death (D) in GBM-GN patients, according to ANCA status at GBM-GN diagnosis. Unadjusted Kaplan-Meier curves are shown using data from the 66 subjects with ANCA status available at diagnosis.

Abbreviations: GBM-GN, glomerular basement membrane disease-associated glomerulonephritis.

Supplementary Figure 1

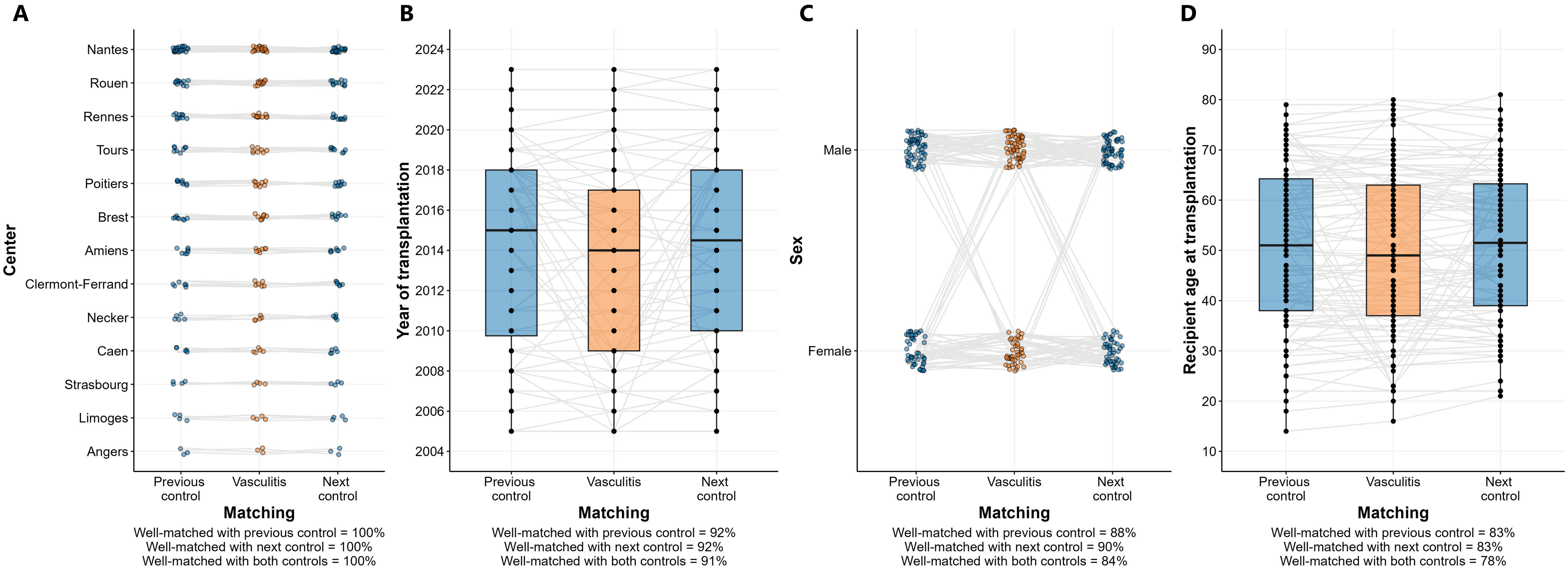

Supplementary Figure 2

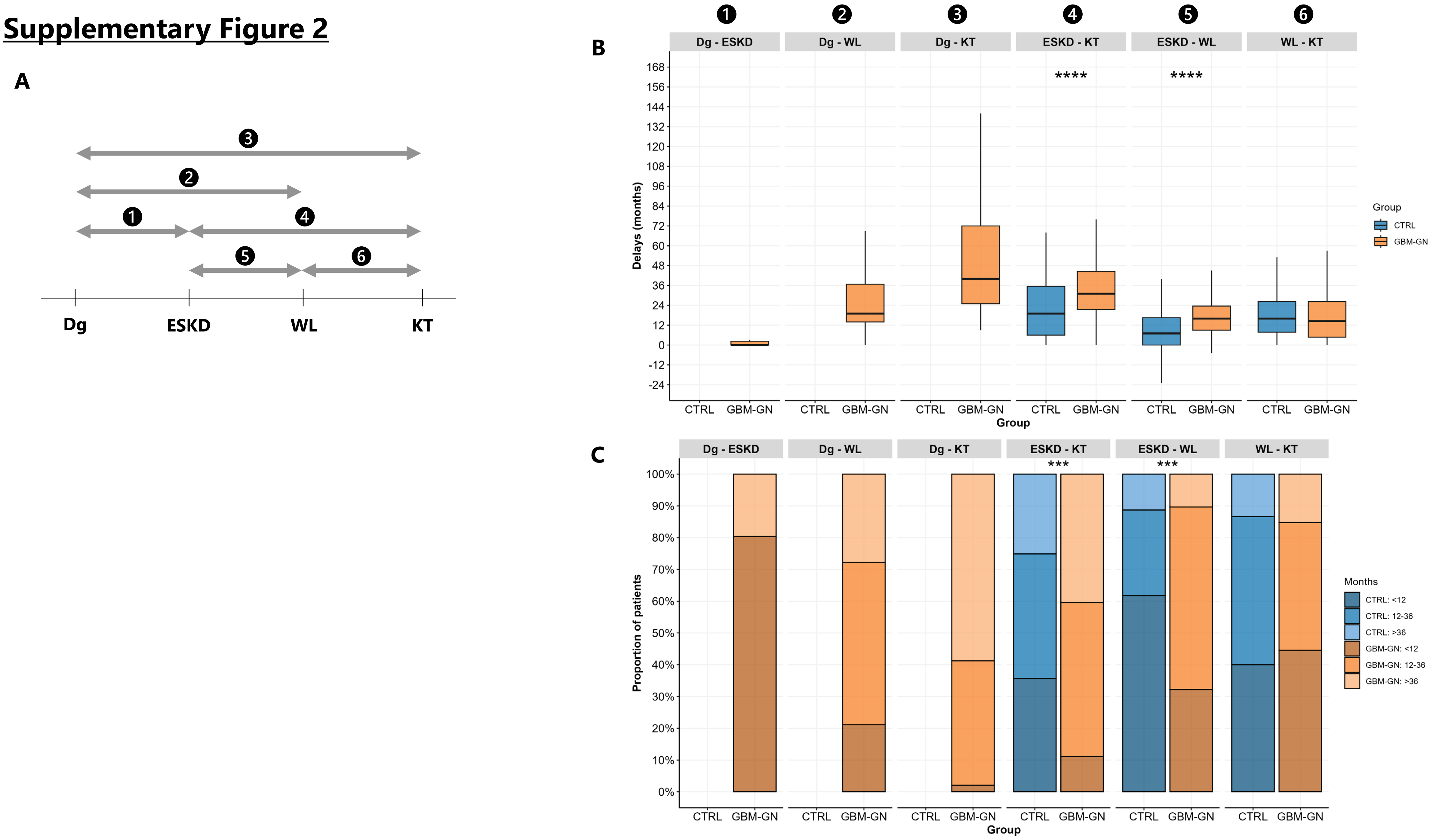

Supplementary Figure 3

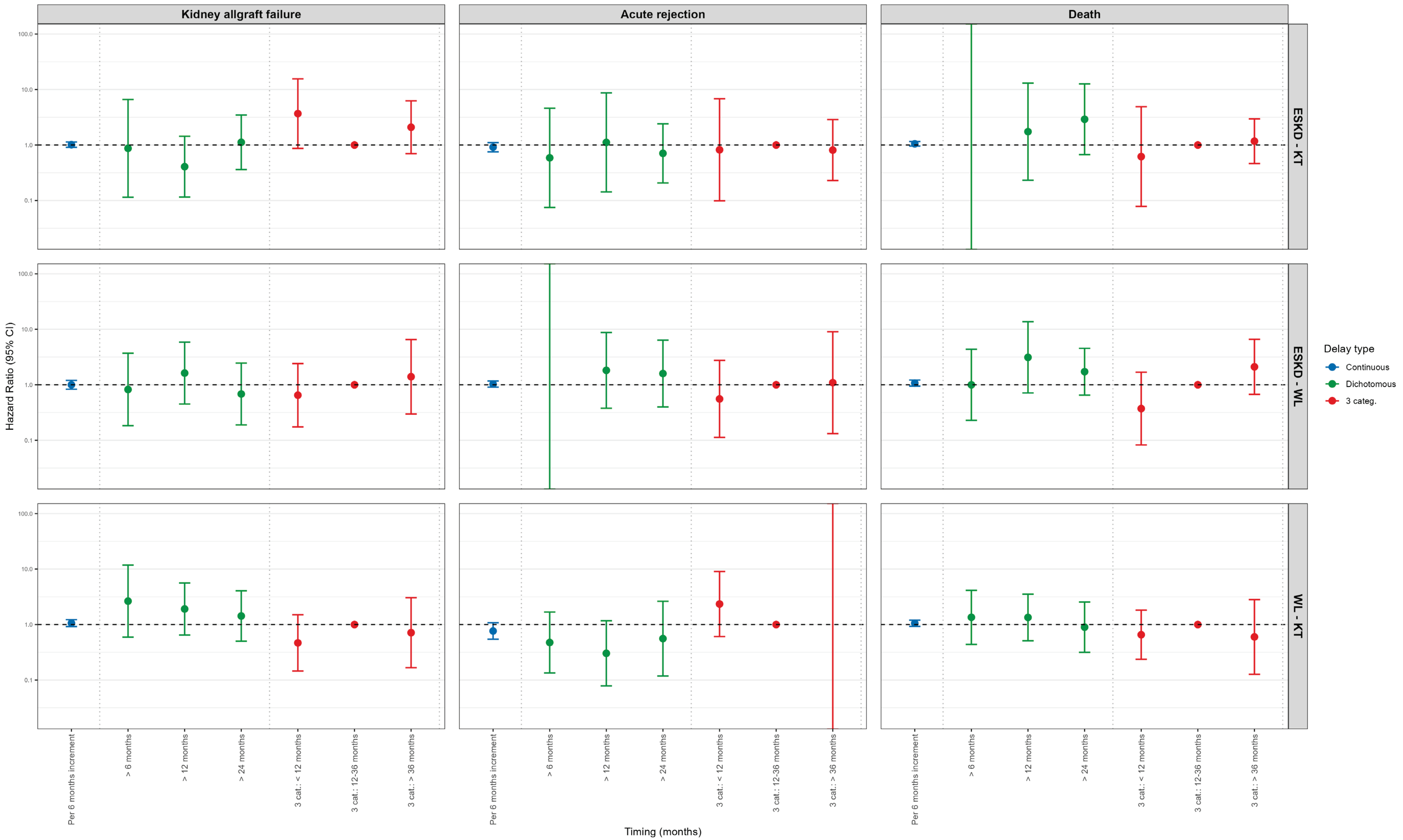

Supplementary Figure 4

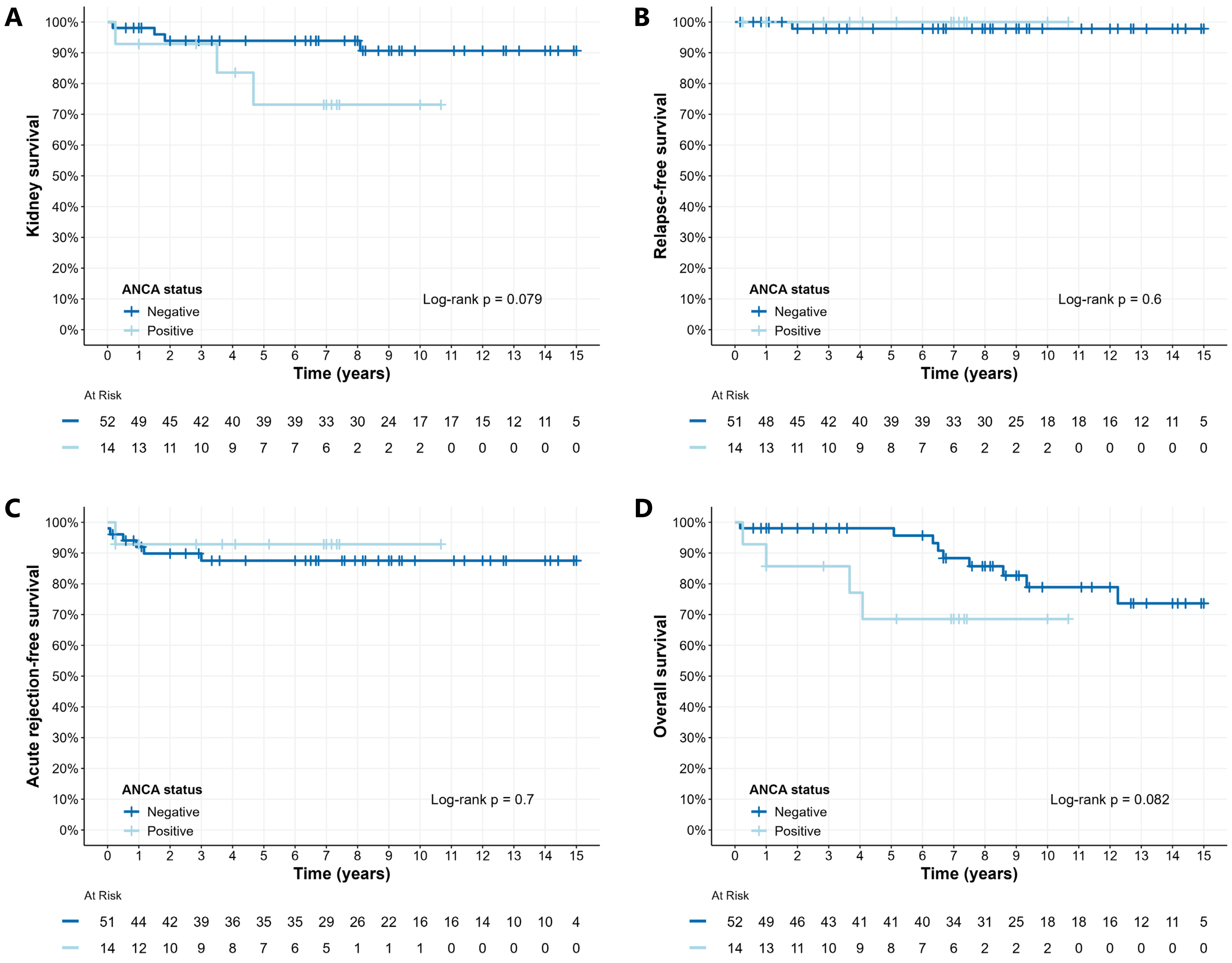

Supplementary Table S1 – Factors associated with DGF (in the GBM-GN cohort)

Abbreviations: ABMR, antibody-mediated rejection; ADPKD, autosomal dominant polycystic kidney disease; AR, acute rejection; BMI, body mass index; DGF, delayed graft function; EIA, enzyme immunoassays; IIF, indirect immunofluorescence; KRT, kidney replacement therapy; KT, kidney transplantation; PRA, panel reactive antibody; TCMR, T-cell mediated rejection. "-" means not applicable. When there was not enough data in some subgroup, Cox regression model could not converge (result is given as "Inf."). * Anti-GBM serological status at the time of KT encompass both IIF and EIA methods.

Supplementary Table S2 – Factors associated with graft failure (in the whole cohort)

Abbreviations: ABMR, antibody-mediated rejection; ADPKD, autosomal dominant polycystic kidney disease; AR, acute rejection; BMI, body mass index; DGF, delayed graft function; EIA, enzyme immunoassays; IIF, indirect immunofluorescence; KRT, kidney replacement therapy; KT, kidney transplantation; PRA, panel reactive antibody; TCMR, T-cell mediated rejection. "-" means not applicable. When there was not enough data in some subgroup, Cox regression model could not converge (result is given as "Inf.").

Supplementary Table S3 – Factors associated with acute rejection (in the whole cohort)

Abbreviations: ABMR, antibody-mediated rejection; ADPKD, autosomal dominant polycystic kidney disease; AR, acute rejection; BMI, body mass index; DGF, delayed graft function; EIA, enzyme immunoassays; IIF, indirect immunofluorescence; KRT, kidney replacement therapy; KT, kidney transplantation; PRA, panel reactive antibody; TCMR, T-cell mediated rejection. "-" means not applicable. When there was not enough data in some subgroup, Cox regression model could not converge (result is given as "Inf.").

Supplementary Table S4 – Factors associated with death (in the whole cohort)

Abbreviations: ABMR, antibody-mediated rejection; ADPKD, autosomal dominant polycystic kidney disease; AR, acute rejection; BMI, body mass index; DGF, delayed graft function; EIA, enzyme immunoassays; IIF, indirect immunofluorescence; KRT, kidney replacement therapy; KT, kidney transplantation; PRA, panel reactive antibody; TCMR, T-cell mediated rejection. "-" means not applicable. When there was not enough data in some subgroup, Cox regression model could not converge (result is given as "Inf.").

Supplementary Table S5 – Distribution of delays between diagnosis, ESKD, waitlisting and kidney transplantation

Supplementary Table 1 – Factors associated with DGF (in the GBM-GN cohort)

	Univariable					Multivariable (simplified)				
**Baseline characteristics**	**N**	**Event N**	**OR**	**95% CI**	**p-value**	**N**	**Event N**	**OR**	**95% CI**	**p-value**
Male sex (vs female)	97	21	2.73	0.96, 9.04	0.074					
BMI (kg/m^2)	96	21	0.95	0.83, 1.08	0.4					
Hypertension (vs no)	97	21	0.92	0.31, 3.14	0.9					
Diabetes (vs no)	97	21	0.58	0.03, 3.70	0.6					
**Presentation at vasculitis diagnosis**										
Age (years)	94	21	1.02	1.00, 1.05	0.11					
Kidney involvement (vs no)	97	21	Inf.	Inf.	-					
Lung involvement (vs no)	79	18	0.72	0.23, 2.12	0.6					
**Immunological findings**										
Presence of anti-GBM abs (vs no)	82	17	Inf.	Inf.	-					
Presence of ANCA (vs no)	65	13	1.87	0.44, 7.11	0.4					
**Kidney transplantation**										
**Status at KT**										
Age (years)	97	21	1.03	1.00, 1.06	0.10					
First transplantation	97	21	1.24	0.35, 5.82	0.8					
Preemptive transplantation	97	21	Inf.	Inf.	-					
Calculated PRA	69	16	1.0	0.98, 1.01	0.5					
Serological status (positive vs negative)	48	12	1.55	0.07, 17.7	0.7					
**Transplantation procedure**										
Donor age (years)	89	18	1.04	1.01, 1.08	**0.027**	89	18	1.04	1.01, 1.08	**0.022**
Deceased donor (vs no)	97	21	Inf.	Inf.	-					
Donor creatinine (per 50 µmol/L increment)	87	20	1.00	0.99, 1.02	0.6					
Cold ischemia time (hours)	96	21	1.10	1.02, 1.20	**0.023**	89	18	1.12	1.03, 1.23	**0.013**
HLA-mismatches (total)	95	21	0.76	0.56, 1.01	0.065					
HLA-A	95	21	0.48	0.21, 1.04	0.068					
HLA-B	95	21	0.63	0.30, 1.32	0.2					
HLA-DR	95	21	0.69	0.33, 1.39	0.3					
HLA-DQ	80	19	0.60	0.25, 1.37	0.2					
**Immunosuppressive regimen**										
Induction therapy										
Anti-thymocyte globulin (vs no)	97	21	1.47	0.55, 3.93	0.4					
Basiliximab (vs no)	97	21	0.84	0.32, 2.25	0.7					
Prednisone (vs no)	95	21	1.76	0.28, 34.4	0.6					
Maintenance regimen										
Calcineurin inhibitors (vs no)	97	21	Inf.	Inf.	-					
Mycophenolic acid (vs no)	97	21	Inf.	Inf.	-					
Prednisone (vs no)	94	19	0.64	0.19, 2.57	0.5					
mTOR inhibitors (vs no)	96	20	1.48	0.37, 4.99	0.5					
Azathioprine (vs no)	96	20	0.61	0.03, 3.90	0.7					

Supplementary Table 2 – Factors associated with graft failure (in the whole cohort)

	Univariable					Multivariable (simplified)				
	**N**	**Event N**	**HR**	**95% CI**	**p-value**	**N**	**Event N**	**HR**	**95% CI**	**p-value**
GBM-GN (vs CTRL)	299	54	0.79	0.45, 1.41	0.4	154	20	1.31	0.49, 3.47	0.6
**Baseline characteristics**										
Male sex (vs female)	299	54	0.82	0.48, 1.40	0.5					
BMI (kg/m^2)	297	52	1.03	0.96, 1.10	0.4					
Hypertension (vs no)	287	49	0.84	0.42, 1.69	0.6					
Diabetes (vs no)	297	53	0.47	0.15, 1.52	0.2					
**Kidney transplantation**										
**Status at KT**										
Age (years)	299	54	1.01	0.99, 1.03	0.4					
First transplantation	298	54	0.51	0.28, 0.94	**0.031**					
Preemptive transplantation	299	54	0.66	0.26, 1.67	0.4					
Calculated PRA	205	30	1.01	1.00, 1.02	**0.047**	154	20	1.01	1.00, 1.02	0.048
**Transplantation procedure**										
Donor age (years)	281	51	1.03	1.01, 1.05	**0.001**	154	20	1.04	1.00, 1.08	**0.034**
Deceased donor (vs no)	296	53	2.92	0.91, 9.39	0.072					
Donor creatinine (per 50 µmol/L increment)	261	45	1.07	0.81, 1.41	0.6					
Cold ischemia time (hours)	292	50	1.05	1.01, 1.08	**0.009**					
HLA-mismatches (total)	295	51	1.15	0.96, 1.37	0.13					
HLA-A	295	51	1.19	0.78, 1.82	0.4					
HLA-B	295	51	0.81	0.55, 1.20	0.3					
HLA-DR	295	51	1.19	0.80, 1.77	0.4					
HLA-DQ	246	44	1.80	1.16, 2.80	**0.009**	154	20	2.43	1.26, 4.70	**0.008**
**Immunosuppressive regimen**										
Induction therapy										
Anti-thymocyte globulin (vs no)	297	52	2.11	1.22, 3.64	**0.007**					
Basiliximab (vs no)	296	52	0.52	0.30, 0.89	**0.018**					
Prednisone (vs no)	296	53	Inf.	Inf.	-					
Maintenance regimen										
Calcineurin inhibitors (vs no)	298	53	0.29	0.04, 2.13	0.2					
Mycophenolic acid (vs no)	297	53	0.64	0.16, 2.63	0.5					
Prednisone (vs no)	281	44	1.04	0.53, 2.02	>0.9					
mTOR inhibitors (vs no)	293	50	0.66	0.26, 1.67	0.4					
Azathioprine (vs no)	292	50	0.67	0.21, 2.15	0.5					
**Outcomes after KT**										
DGF (vs no)	285	48	2.60	1.43, 4.75	**0.002**	154	20	3.78	1.45, 9.84	**0.007**
Allograft failure (vs no)										
Rejection (vs no)	293	53	3.27	1.90, 5.64	**<0.001**	154	20	4.52	1.60, 12.7	**0.004**
Acute rejection (AR) (vs no)	293	54	2.35	1.31, 4.21	**0.004**					
TCMR (vs no)	293	54	1.63	0.77, 3.46	0.2					
ABMR (vs no)	292	53	4.52	2.26, 9.04	**<0.001**					
Mixed AR (vs no)	299	54	4.09	1.62, 10.3	**0.003**					
Chronic rejection	290	53	5.86	3.06, 11.2	**<0.001**					
Death	-	-	-	-	-					

Supplementary Table 3 – Factors associated with acute rejection (in the whole cohort)

	Univariable					Multivariable (simplified)				
	N	Event N	HR	95% CI	p-value	N	Event N	HR	95% CI	p-value
GBM-GN (vs CTRL)	292	45	0.63	0.32, 1.24	0.2	281	43	0.51	0.25, 1.02	0.055
**Baseline characteristics**										
Male sex (vs female)	292	45	1.08	0.60, 1.96	0.8					
BMI (kg/m^2)	290	44	1.00	0.93, 1.07	0.9					
Hypertension (vs no)	281	43	0.28	0.15, 0.52	**<0.001**	281	43	0.32	0.17, 0.61	**<0.001**
Diabetes (vs no)	291	44	0.62	0.22, 1.72	0.4					
**Kidney transplantation**										
**Status at KT**										
Age (years)	292	45	0.97	0.95, 0.99	**<0.001**	281	43	0.97	0.95, 0.99	**0.003**
First transplantation	291	45	0.76	0.37, 1.58	0.5					
Preemptive transplantation	292	45	1.02	0.43, 2.41	>0.9					
Calculated PRA	201	28	1.00	0.99, 1.01	0.7					
**Transplantation procedure**										
Donor age (years)	274	44	0.98	0.97, 1.00	0.077					
Deceased donor (vs no)	289	44	0.82	0.36, 1.83	0.6					
Donor creatinine (per 50 µmol/L increment)	255	41	1.14	0.87, 1.48	0.3					
Cold ischemia time (hours)	285	43	0.97	0.93, 1.01	0.14					
HLA-mismatches (total)	289	44	1.16	0.96, 1.40	0.12					
HLA-A	289	44	1.01	0.64, 1.58	>0.9					
HLA-B	289	44	1.13	0.72, 1.78	0.6					
HLA-DR	289	44	1.26	0.82, 1.94	0.3					
HLA-DQ	240	38	1.54	0.97, 2.46	0.068					
**Immunosuppressive regimen**										
Induction therapy										
Anti-thymocyte globulin (vs no)	290	44	0.67	0.35, 1.31	0.2					
Basiliximab (vs no)	289	44	1.40	0.74, 2.64	0.3					
Prednisone (vs no)	290	43	0.53	0.21, 1.34	0.2					
Maintenance regimen										
Calcineurin inhibitors (vs no)	291	44	Inf.	Inf.	-					
Mycophenolic acid (vs no)	290	44	Inf.	Inf.	-					
Prednisone (vs no)	274	38	2.02	0.84, 4.82	0.12					
mTOR inhibitors (vs no)	286	42	0.62	0.22, 1.73	0.4					
Azathioprine (vs no)	285	42	0.29	0.04, 2.09	0.2					
**Outcomes after KT**										
DGF (vs no)	278	40	1.10	0.51, 2.39	0.8					
Allograft failure (vs no)	-	-	-	-	-					
Rejection (vs no)	-	-	-	-	-					
Acute rejection (AR) (vs no)	-	-	-	-	-					
TCMR (vs no)	-	-	-	-	-					
ABMR (vs no)	-	-	-	-	-					
Mixed AR (vs no)	-	-	-	-	-					
Chronic rejection	-	-	-	-	-					
Death	-	-	-	-	-					

Supplementary Table 4 – Factors associated with death (in the whole cohort)

	Univariable					Multivariable (simplified)				
	**N**	**Event N**	**HR**	**95% CI**	**p-value**	**N**	**Event N**	**HR**	**95% CI**	**p-value**
GBM-GN (vs CTRL)	300	49	1.12	0.63, 1.99	0.7	268	45	0.85	0.46, 1.58	0.6
**Baseline characteristics**										
Male sex (vs female)	300	49	1.88	1.0, 3.54	0.052	268	45	2.11	1.08, 4.13	**0.029**
BMI (kg/m^2)	298	49	1.04	0.99, 1.10	0.15					
Hypertension (vs no)	288	47	1.16	0.54, 2.49	0.7					
Diabetes (vs no)	298	49	1.19	0.51, 2.81	0.7					
**Kidney transplantation**										
**Status at KT**										
Age (years)	300	49	1.08	1.05, 1.10	**<0.001**	268	45	1.05	1.01, 1.08	**0.006**
First transplantation	299	49	0.80	0.40, 1.60	0.5					
Preemptive transplantation	300	49	0.15	0.02, 1.06	0.057	268	45	0.14	0.02, 1.08	0.059
Calculated PRA	206	32	0.99	0.98, 1.00	0.2					
**Transplantation procedure**										
Donor age (years)	282	47	1.07	1.05, 1.09	**<0.001**	268	45	1.03	1.00, 1.06	0.062
Deceased donor (vs no)	297	49	1.50	0.59, 3.80	0.4					
Donor creatinine (per 50 µmol/L increment)	262	41	0.69	0.41, 1.17	0.2					
Cold ischemia time (hours)	293	48	1.02	0.99, 1.05	0.2					
HLA-mismatches (total)	296	48	1.01	0.85, 1.20	>0.9					
HLA-A	296	48	1.00	0.65, 1.54	>0.9					
HLA-B	296	48	0.97	0.63, 1.47	0.9					
HLA-DR	296	48	1.05	0.69, 1.59	0.8					
HLA-DQ	247	38	1.03	0.65, 1.66	0.9					
**Immunosuppressive regimen**										
Induction therapy										
Anti-thymocyte globulin (vs no)	298	49	0.76	0.41, 1.42	0.4					
Basiliximab (vs no)	297	49	1.41	0.78, 2.57	0.3					
Prednisone (vs no)	297	48	1.37	0.42, 4.40	0.6					
Maintenance regimen										
Calcineurin inhibitors (vs no)	299	49	Inf.	Inf.	-					
Mycophenolic acid (vs no)	298	49	0.54	0.13, 2.23	0.4					
Prednisone (vs no)	282	45	1.12	0.58, 2.17	0.7					
mTOR inhibitors (vs no)	294	47	1.07	0.48, 2.40	0.9					
Azathioprine (vs no)	293	46	0.99	0.35, 2.77	>0.9					
**Outcomes after KT**										
DGF (vs no)	286	47	2.72	1.51, 4.90	**<0.001**	268	45	1.70	0.91, 3.17	0.10
Allograft failure (vs no)	299	48	1.39	0.72, 2.67	0.3					
Rejection (vs no)	294	46	0.65	0.29, 1.45	0.3					
Acute rejection (AR) (vs no)	294	46	0.34	0.10, 1.09	0.070					
TCMR (vs no)	294	46	0.76	0.27, 2.11	0.6					
ABMR (vs no)	293	46	Inf.	Inf.	-					
Mixed AR (vs no)	300	49	Inf.	Inf.	-					
Chronic rejection	291	46	1.91	0.76, 4.85	0.2					
Death	-	-	-	-	-					

Supplementary Table 5 – Distribution of delays between diagnosis, ESKD, waitlisting and kidney transplantation

**Delays**	**N**	**GBM-GN**, N = 100	**CTRL**, N = 200	**p-value**
Follow-up (time from KT to last visit)	300	97 (46, 145)	76 (42, 123)	0.14
**GBM-GN diagnosis to ESKD (months)**	92	0 (0, 2)	-	
More than 6 months	92	21 (23%)	-	
More than 12 months	92	18 (20%)	-	
More than 24 months	92	18 (20%)	-	
3 categories	92			
<12 months		74 (80%)	-	
12-36 months		0 (0%)	-	
>36 months		18 (20%)	-	
**GBM-GN diagnosis to KT (months)**	97	40 (25, 72)	-	
More than 6 months	97	97 (100%)	-	
More than 12 months	97	95 (98%)	-	
More than 24 months	97	79 (81%)	-	
3 categories	97			
<12 months		2 (2.1%)	-	
12-36 months		38 (39%)	-	
>36 months		57 (59%)	-	
**GBM-GN diagnosis to waitlisting**	90	19 (14, 37)	-	
More than 6 months	90	88 (98%)	-	
More than 12 months	90	71 (79%)	-	
More than 24 months	90	38 (42%)	-	
3 categories	90			
<12 months		19 (21%)	-	
12-36 months		46 (51%)	-	
>36 months		25 (28%)	-	
**ESKD to KT**	298	31 (22, 45)	19 (6, 36)	**<0.001**
More than 6 months	298	93 (94%)	150 (75%)	**<0.001**
More than 12 months	298	88 (89%)	128 (64%)	**<0.001**
More than 24 months	298	69 (70%)	82 (41%)	**<0.001**
3 categories	298			**<0.001**
<12 months		11 (11%)	71 (36%)	
12-36 months		48 (48%)	78 (39%)	
>36 months		40 (40%)	50 (25%)	
**ESKD to waitlisting**	202	16 (9, 24)	7 (0, 17)	**<0.001**
More than 6 months	202	75 (86%)	61 (53%)	**<0.001**
More than 12 months	202	59 (68%)	44 (38%)	**<0.001**
More than 24 months	202	22 (25%)	17 (15%)	0.061
3 categories	202			**<0.001**
<12 months		28 (32%)	71 (62%)	
12-36 months		50 (57%)	31 (27%)	
>36 months		9 (10%)	13 (11%)	
**Waitlisting to KT**	272	15 (5, 26)	16 (8, 26)	0.3
More than 6 months	272	68 (74%)	147 (82%)	0.14
More than 12 months	272	51 (55%)	108 (60%)	0.5
More than 24 months	272	27 (29%)	57 (32%)	0.7
3 categories	272			0.6
<12 months		41 (45%)	72 (40%)	
12-36 months		37 (40%)	84 (47%)	
>36 months		14 (15%)	24 (13%)	
